# Demonstration of Tunable Steering and Multiplexing of Two 28 GHz Data Carrying Orbital Angular Momentum Beams Using Antenna Array

**DOI:** 10.1038/srep37078

**Published:** 2016-11-11

**Authors:** Guodong Xie, Zhe Zhao, Yan Yan, Long Li, Yongxiong Ren, Nisar Ahmed, Yinwen Cao, Asher J. Willner, Changjing Bao, Zhe Wang, Cong Liu, Morteza Ziyadi, Shilpa Talwar, Soji Sajuyigbe, Solyman Ashrafi, Moshe Tur, Andreas F. Molisch, Alan E. Willner

**Affiliations:** 1 Department of Electrical Engineering, University of Southern California, Los Angeles, CA 90089, USA; 2 Intel Labs, Intel Corporation, Santa Clara, CA 95054, USA; 3 NxGen Partners, Dallas, TX 75219, USA; 4 School of Electrical Engineering, Tel Aviv University, Ramat Aviv 69978, Israel

## Abstract

In line-of-sight communication systems, accurate alignment between the transmitter and receiver is important to guarantee sufficient signal power at the receiver. Such alignment is even more important for orbital angular momentum (OAM) multiplexing systems since misalignment between the transmitter and receiver may cause crosstalk among channels. In this paper, we demonstrate the simultaneous generation and tunable steering of two OAM beams utilising a custom-designed circular antenna array at 28 GHz. We achieve a steering angle of up to 35 degrees from the antenna array normal. We find that (*i*) the steering angle of the generated OAM beams is limited by the emitting angle of the antenna elements, and (*ii*) a larger steering angle may degrade the mode purity of the generated OAM beams as well as induce inter-symbol-interference to each of the individual channels. Moreover, we demonstrate the transmission of two 1-Gbaud quadratic phase shift keying (QPSK) signal over the two steerable OAM beams with both multiplexed channels achieved bit error rates (BERs) of <3.8 × 10^−3^.

There is continual interest to increase both the capacity and spectral efficiency in free-space radio frequency (RF) line-of-sight communication systems^1^,^2^,^3^. One technique for such an increase is to multiplex multiple data-carrying beams so that they can be transmitted through a single transmitter/receiver aperture pair. If each beam occupies a unique spatial mode in an orthogonal basis set, then channel multiplexing and transmission could occur with little inherent crosstalk and mode coupling. Such mode-division-multiplexing (MDM) has been demonstrated in free-space line-of-sight communication links using multiple orbital angular momentum (OAM) modes in RF^4^,^5^,^6^,^7^,^8^,^9^,^10^ and optical regiems^11^,^12^.

An OAM beam has a phase front of exp(*jℓθ*) that “twists” in a helical fashion as it propagates, where *ℓ* is the OAM order (*ℓ* = 0, ±1, ±2, …) and *θ* is the azimuthal angle. Theoretically, the different twisting rates ensure the orthogonality among OAM beams with different *ℓ* values^13^,^14^. OAM beams could be generated in several ways in the RF domain, including passing a conventional Gaussian beam through a spiral phase plate (SPP)^15^,^16^ and directly generating one or multiple OAM beams from a circular array of antenna elements^17^,^18^,^19^.

In line-of-sight communication systems, accurate alignment between the transmitter and receiver is important to guarantee sufficient signal power at the receiver. Such alignment is even more important for OAM multiplexing systems since misalignment between the transmitter and receiver may cause crosstalk among channels^20^. Therefore, it would be desirable to design a transmitter that can not only generate OAM beams but also steer the beams for active pointing. Previous reports have experimentally shown the ability to steer an optical OAM beam by shaping an input single Gaussian beam with a fork hologram that combines a spiral phase and a tilted phase front (e.g., a grating)^21^,^22^. However, few reports have shown the steering of RF OAM beams using a circular antenna array^23^,^24^.

In this paper, we demonstrate the simultaneous generation and tunable steering of multiple OAM beams at a 28-GHz carrier frequency using a circular array of antenna elements. Since beam steering and OAM generation both rely on delays among the multiple beams, by tailoring the delays among different elements, two OAM beams are generated with a variable steering angle of up to 35 degrees. We investigated the effects of the beam steering on the quality of the generated OAM beams. Moreover, we use the steerable OAM beams to establish a 4 Gbit/s communication link, with each OAM beam carrying a 1-Gbaud quadratic-phase-shift-keying (QPSK) signal.

Figure 1a shows the principle of the simultaneous generation and steering of multiple OAM beams using a single antenna array. An RF sine-wave generator of frequency *ω*_*c*_ is shared by *M* modulators (the case of *M* = 2 is shown in the figure), which are fed by *M* data streams, {*A*_*m*_(*t*), *m* = 1, …, *M*}, to produce *M* modulated signals with the same carrier frequency: {*A*_*m*_(*t*) exp(*jω*_*c*_*t*), *m* = 1, …, *M*}. Each of the *M* signals is then split into *N* (the number of antenna elements in the array, *N* = 8 is shown in the figure) branches, where each of the *M·N* branch is assigned a pre-determined time delay *τ*_*mn*_, *m* = 1, …, *M, n* = 1, …*N.* Each delay *τ*_*mn*_ is the sum of two terms: *τ*_*mn*_ = *τ*_*mn*,*S*_ + *τ*_*mn*,*O*_. The first term is designed for the steering and the second term is designed for the generation of an OAM beam of order *ℓ*_*m*_. The steering part is given by the well-known equation^3^,^25^: *τ*_*mn*,*S*_ = *R*·cos*θ*_*mn*_·sin(*α*_*m*_)/*c*, where *R*, the array radius, is the distance from the array centre to the circularly placed antenna elements; *α*_*m*_ is the beam steering angle of the *m*-th beam;*θ*_*mn*_ = 2*π*(*n* − 1)/*N* is the azimuthal angle of *n*-th antenna element *α*_*m*_ from a plane, ∏_*m*_, formed by a line pointing at the steering direction and the the antenna array normal; and *c* is the propagation speed of the electromagnetic wave in free space. The generation of the OAM beam of order *ℓ*_*m*_ requires azimuthal phase delays, given by *φ*_*m*_ = *ℓ*_*m*_*θ*_*mn*_, which, at carrier frequency *ω*_*c*_, can be achieved by the time delays *τ*_*mn*,*O*_ = *φ*_*m*_/*ω*_*c*_ = *ℓ*_*m*_*θ*_*mn*_/*ω*_*c*_. Multiple OAM beams of orders {*ℓ*_*m*_, *m* = 1, …*M*}, can be simultaneously generated and at the same time individually steered to angles {*α*_*m*_, *m* = 1, …*M*}, if the *n*-th element of the antenna array is fed with the superposition: 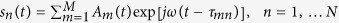.

As shown in Fig. 1a,c, such a superposition is implemented using *N* field combiners, each with *M* inputs and a single summing output, where: (*i*) The output of the *n*-th combiner is connected to the *n*-th element of the antenna array; (*ii*) The *m*-th inputs of the *N* combiners are fed by the *N* delayed signals associated with the *m*-th beam. Figure 1c shows our proposed design for the case of *M* = 2 beams and *N* = 8 antenna elements. The required time delays are implemented by “trombone-like” tunable time delay lines. Thus, for the generation and steering of beam #1, the time delays {*τ*_11_, *τ*_12_, *τ*_13_, *τ*_14_, *τ*_15_, *τ*_16_, *τ*_17_, *τ*_18_} are applied to the correspondingly designated 8 delays in Fig. 1(c), while beam #2 generated and steered by applying {*τ*_21_, *τ*_22_, *τ*_23_, *τ*_24_, *τ*_25_, *τ*_26_, *τ*_27_, *τ*_28_} to the other group of 8 delay lines, which then feed the other input ports of the 8 field combiners. Previous reports have indicated that the order of OAM beams that could be generated from a ring antenna array with *N* elements obeys 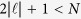
4,19,26. As an example, with 8 antenna elements used in our design, the order of the generated OAM beams obeys 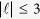.

## Results

We first investigate the generation of a single unmodulated OAM + 1 beam using the designed antenna array. Figure 2a shows the intensity profile of the generated OAM + 1 beam at the distance of 1.2 m from the antenna array (see the Method section for the measurement and simulation approaches) with an array radius *R* of 4.5 cm. The 8 array elements are excited with phase delays of 0, π/4, π/2, 3π/4, π, 5π/4, 3π/2 and 7π/4. Our results agree well with the previous studies in the RF domain^17^,^18^,^19^ and in the optical domain^27^. Besides, the beam’s phase distribution is characterized through its interference (see Fig. 2b), with a regular Gaussian beam^28^ (see the Method section for the measurement approach). The clean rotating arm is in good match with the simulation result.

In addition to the near field and far field intensity profiles of the generated OAM + 1 beam^27^, we also investigate the evolution of the beam’s intensity profiles at different distances. Figure 2c1–c5 depict the intensity profiles of the generated OAM + 1 beam at various ranges from 0.4 to 1.2 m. Our experimental results agree well with the simulation predictions. At a very short distance, the ring shape of the generated OAM beam is somewhat unclear, while after some propagation, the intensity profile evolves to a better ring shape. Due to the experimental limitation, we only measure the beam evolution at a distance up to 1.2 m. The distance that the generated OAM beam could propagate is determined by the divergence of the beam and its diffraction limit^28^.

An OAM beam diverges faster than a regular Gaussian beam with the same beam waist, while the receiver for the communication system might have a limited aperture size. Therefore, taking into account the divergence of the generated OAM beams is imperative for the system design. As expected, we find that the OAM beam’s divergence is inversely proportional to the array radius *R*. Figure 3a–c show the intensity profiles of the generated OAM+1 beam using an 8-element antenna array after a 1.2-m propagation, when *R* is 4.5 cm, 5 cm, and 6 cm, respectively. Figure 3d shows the simulated and measured beam sizes as a function of array radius *R*. Since the waist of the generated beam is proportional to the array radius, larger *R* would result in a smaller far-field pattern. Therefore, for a practical implementation, the array radius needs to be properly designed considering the divergence of the generated OAM beams and the link distance. An additional study of the effects of the number of antenna elements per array and their spatial arrangement on the quality of the generated OAM beams appears in the Supplementary Section.

**Figure 1:**
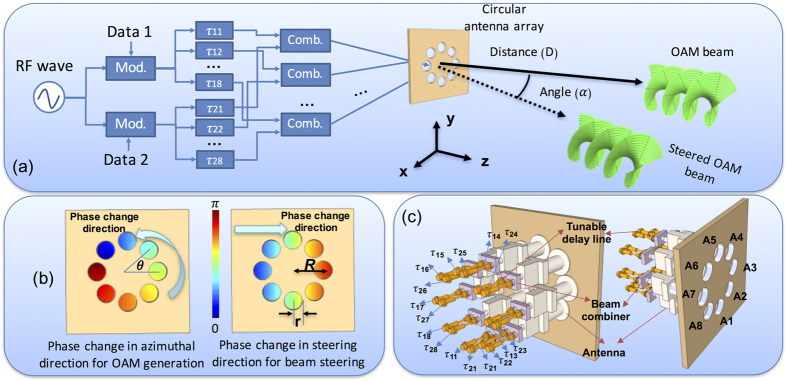
Principle of the Technique for the Simultaneous Generation and Steering of Multiple OAM Beams. (**a**) The concept of using an antenna array for the simultaneous generation and steering of multiple OAM beams. x: horizontal direction; y: vertical direction; z: direction perpendicular to the antenna array plane; Mod: modulator; Comb.: beam combiner; *τ*: time delay. (**b**) The relative phase change in the azimuthal direction for OAM generation and the relative phase for horizontal beam steering. (**c**) The design of an antenna array for the generation and steering of two OAM beams. τ_11_, τ_12_, τ_13_, τ_14_, τ_15_, τ_16_, τ_17_ and τ_18_, are designed for the generation and steering of one OAM beam, while τ_21_, τ_22_, τ_23_, τ_24_, τ_25_, τ_26_, τ_27_ and τ_28_ are designed for the generation and steering of another OAM beam).

**Figure 2:**
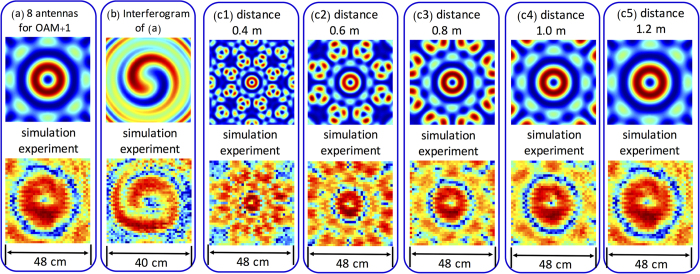
Simulation and Experiment Results of the Generation of One OAM Beam. (**a**) The intensity profile of the OAM + 1 beam generated from an array of 8 antenna elements at 1.2 m. (**b**) The interference pattern of the generated OAM + 1 beam with a Gaussian beam. (c1-c5) The intensity profiles of OAM + 1 beams generated from 8 antennas at 0.4 m, 0.6 m, 0.8 m, 1.0 m and 1.2 m, respectively.

**Figure 3:**
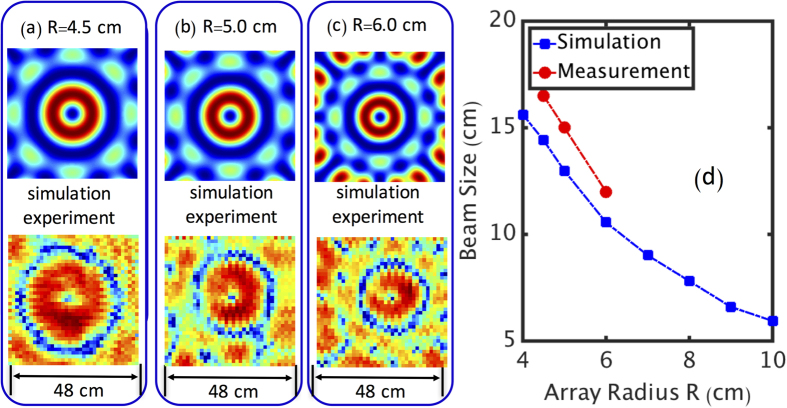
Simulation and Experimental Results of the OAM Beam Generation with Different Array Radii. The intensity profiles of the generated OAM + 1 beams with array radii of (**a**) 4.5 cm, (**b**) 5.0 cm and (**c**) 6.0 cm. (**d**) The simulated and measured beam size at 1.2 m as a function of array radius *R*.

**Figure 4:**
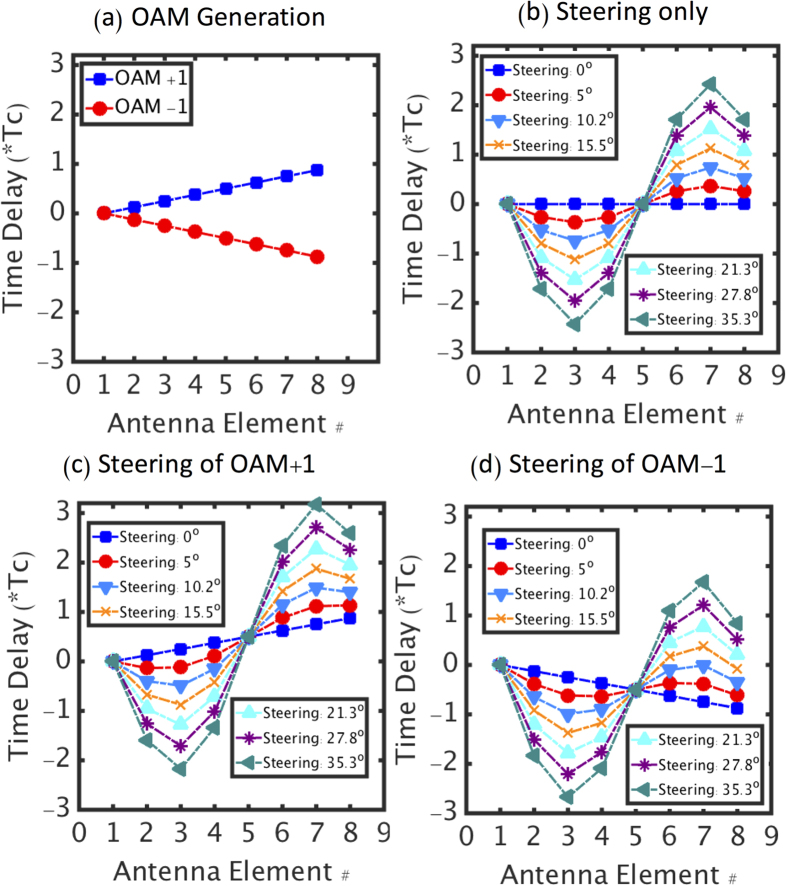
The Design of Time Delay. (**a**) The delays of the 8 antennas elements for the generation of the OAM + 1 and OAM-1 beams. (**b**) The delays for the 8 antennas elements for the steering of the beams. The bottom two panels show the delays applied to the 8 antennas elements for the simultaneous generation and steering of the OAM + 1 (**c**) and OAM-1 beams (**d**). *T*_*c*_: the period of the carrier wave. In practice, only positive delays were implemented so that the element with the most negative delay (A3) was considered a reference, having zero delay, and all other elements were assigned relative delays according to the figures above.

Next, we investigate the simultaneous generation and steering of one, as well as multiple OAM beams using the 8-element antenna array, initially with no data modulation. Figure 4a shows the time delays among the 8 antennas only for the generation of the OAM + 1 and OAM-1 beams (*α*_*m*_ = 0, *m* = 1, 2), where these delays simply increase or decrease monotonically with the azimuthal location of the element. Figure 4b shows the relative time delays for beam steering with various angles without imparting an OAM charge to the beams. Since the steering is designed to be in the horizontal plane, the two elements in each of the pairs {A1, A5}, {A2, A4} and {A6, A8} (Fig. 1c) share the same delay, regardless of the steering angle. Figure 4c shows the combined delays, which would allow the simultaneous generation and steering of the OAM + 1 beam. Figure 4d shows the delays required for the simultaneous generation and steering of the OAM-1 beam. Moreover, the OAM + 1 and OAM-1 beams could be generated and steered simultaneously if the different branches of the two beams are combined using beam combiners in Fig. 1a.

**Figure 5:**
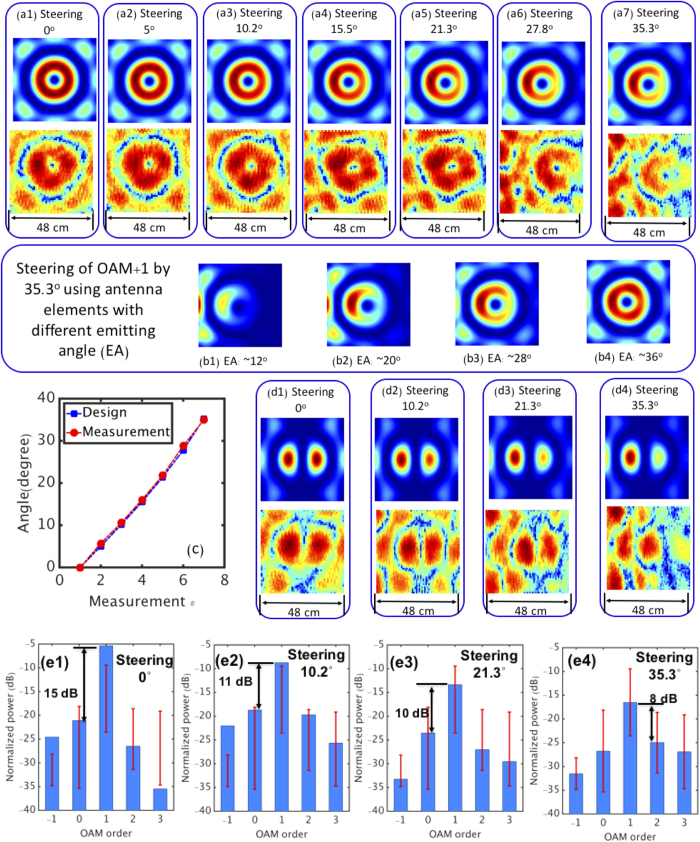
Experimental and Simulation Results of the Simultaneous Generation and Steering of One and Two OAM Beams. (a1-a6) Intensity profiles of the steered OAM + 1 beam with a steering angle of 0°, 5°, 10.2°, 15.5°, 21.3°, 27.8°, and 35.3°, respectively. (b1-b4) Simulation results of the steering of OAM + 1 beam when the antenna elements have different emitting angle (EA). (**c**) Comparison between the designed steering angle and the measured steering angle. (d1–d4) Intensity profiles of the steered superposition of the OAM + 1 beam and OAM-1 beam with steering angles of 0°, 10.2°, 21.3°, and 35.3°, respectively. (e1–e4) The distribution of the received power among several modes when only the OAM + 1 beam is generated with steering angles of 0°, 10.2°, 21.3°, and 35.3°, respectively. The measurement is performed at 1.2 m. The plot represents the average of multiple measurements, while the error bars indicate standard deviations.

Before presenting steering results, it is important to note that while Fig. 4 calls for relative delays in access of 4–5 periods of the carrier (*T*_*c*_ = 2*π*/*ω*_*c*_), our delay-generating RF trombones could only cover a range of 0 to *T*_*c*_. Therefore, we set each of the delays to a value derived from its true delay value *modulo T*_*c*_ (*e.g.*, a delay of 5.5*T*_*c*_ was implemented by a delay of 0.5*T*_*c*_). Since an integer number of periods represent a multiple of 2*π*, this transformation is harmless as long as there is no modulation on the carrier, as in shown Fig. 5 below.

Figure 5a1–a7 show the intensity profiles of the steered OAM + 1 beam (see Method section for the measurement approach) at various steering angles. When increasing the steering angle, distortions are observed in the resulting beam pattern. The divergence angle of the beam emitted from each antenna element is ~28° and the total coverage area of the antenna array is limited by this angle. Therefore, larger steering angle may cause the quality degradation of the steered OAM beam due to the limited coverage area as simulated in Fig. 5b1–b4. Figure 5c shows the comparison between the designed steering angle and measured steering angle, which indicates our steering angle is within the error of less than 1°.

As described above, the OAM + 1 and OAM-1 beams could be simultaneously generated and steered using the same antenna array by feeding each antenna element with the sum of the respective contributions of the two OAM beams (Fig. 1a). Figure 5d1–d4 show the superposition of the OAM + 1 and OAM-1 beams with steering angles of 0°, 10.2°, 21.3°, and 35.3°, respectively, clearly indicating that the designed structure has achieved the simultaneous generation and steering of multiple OAM beams. We note that by properly changing the relative time delays, a steering of −35.3° should also be achievable.

For a communication system, OAM mode purity, characterized by the distribution of the received power among OAM modes other than the transmitted one, is one of the most important factors that affects the channel crosstalk. Figure 5e1–e4 show the mode purity of the generated OAM + 1 beam when the steering angle varies from 0° to 35.3° (see Method section for the measurement approach). The power leaked to neighbouring modes increases as the steering angle varies from 0° to 35.3°. The larger the steering angle of an OAM beam in our setup, the more distortions (Fig. 5a), and consequently, the stronger the leakage to neighbouring modes.

**Figure 6:**
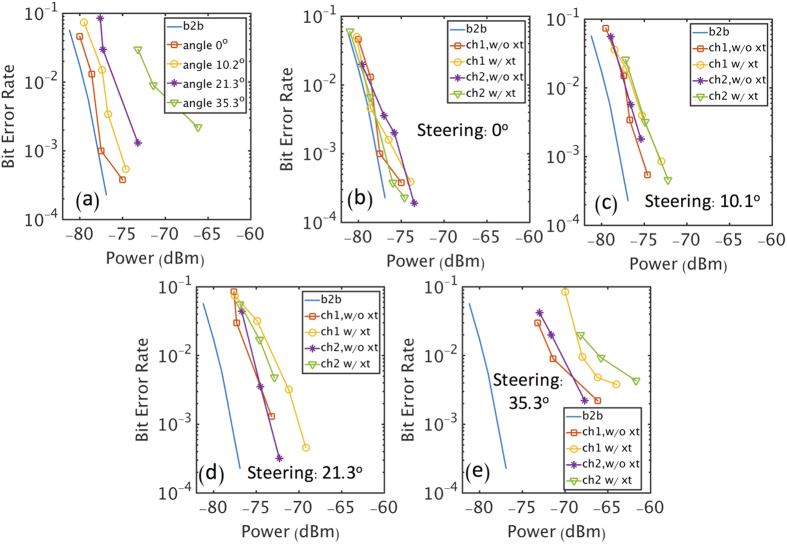
BER Measurement. (a1) BER performance when only one OAM beam is generated and steered with various steering angles. (**b**–**e**) BER performance when one or two OAM beams are transmitted at different steering angles. xt: crosstalk; w/: with; w/o: without; b2b: back to back, where the transmitter is connected to the receiver using a cable.

For the investigation of the bit error rate (BER) performance of the proposed scheme, two 1-GBaud QPSK signal streams were used to modulate the multiplexed and steered OAM + 1 and OAM − 1 beams. After free space propagation of ~1.2 m, the signal was de-multiplexed and recorded by an oscilloscope for BER measurement. As shown in Fig. 6a, as the steering angle increases, the BER goes up for a specific received power even when only one OAM channel is transmitted (no crosstalk). This trend is attributed to the deviation from true time delay steering, introduced by the *modulo* transformation between the true delay value and the implemented one. At maximum steering of 35.3°, the required relative true time delay of 5.5*T*_*c*_ between antenna elements A3 and A7 was implemented by a 0.5*T*_*c*_. This implementation created an unwanted time difference of 5*T*_*c*_ (almost 18% of the baud duration) between the modulation information coming from these two elements, giving rise to inter-symbol interference and higher BER. While deteriorating with increasing steering, even at 35.3° BER performance of 3.8 × 10^–3^ could be achieved.

As shown in Fig. 6b–e, when the steering angle is small, the BER of multiplexing two OAM channels is very close to the case when only a single OAM channel is transmitted due to the low channel crosstalk. When the steering angle is 21.3° and 35.3°, penalties of ~3 dB and ~5 dB are observed for the multiplexing of two channels compared to that with only one channel. However, the system could still achieve a BER below 3.8 × 10^−3^ even at a steering angle of 35.3°.

## Discussion

We have demonstrated the simultaneous generation and steering of multiple OAM beams utilising a custom-designed circular antenna array at 28 GHz. Two OAM beams were generated with a steering angle of up to 35°. The following points are also worth mentioning: (*i*) For optimum performance it is important not only to control the time delays but also to minimize variations among the powers emitted from the different antenna elements. (*ii*) Only horizontal steering was demonstrated. Indeed, the proposed approach can achieve beam steering in any direction, at least within the tested cone of 70° apex angle. (*iii*) The same concept and implementation should work equally well at other RF frequencies. (*iv*) The implemented tunable delay in our experiment was limited to one period of the RF carrier. The inter-symbol interference caused by large steering angles can be reduced when true time delay^29^ operation is achieved by using tunable delay lines what is capable of covering the full required delay range. (*v*) In general, Laguerre-Gaussian (LG) modes represent a complete 2 dimensional modal basis set and can be described by two indices (i.e., an azimuthal index *ℓ* and a radial index *p*), and OAM can exist for LG modes with different *p* values. If only *p* = 0 is used for multiple beams of different *ℓ* values^30^, then this can be considered a subset of the fuller 2 dimensional set of LG modes. However, since OAM can exist for *p*≠0 as well, then the use of different p values and different *ℓ* values can produce a fuller set of modes and theoretically a higher system capacity over a given spatial area^31^,^32^.

## Methods

### The method for the intensity profile measurement the generated beams

**Figure 7:**
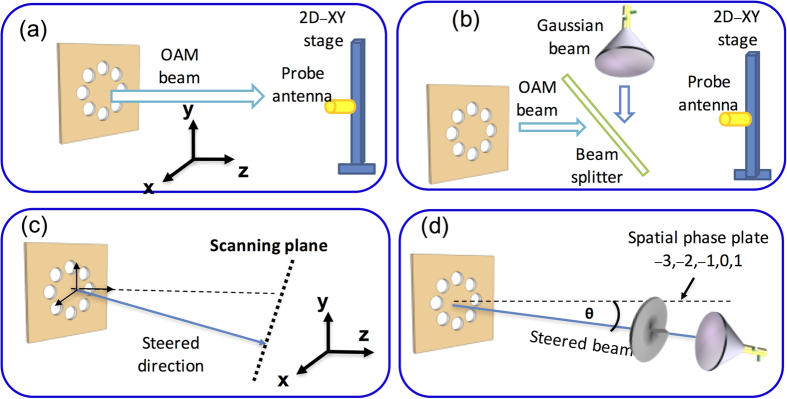
Measurement Design. (**a**) The measurement setup to scan the intensity profile of the generated OAM beams without steering. (**b**) The measurement setup to scan the interference pattern of the generated OAM beams without steering. (**c**) The measurement setup to scan the intensity profiles of the steered OAM beams. (**d**) The measurement setup to test the mode purity of the steered OAM beams.

As shown in Fig. 7a, we measure the intensity profiles of the generated beams using a probe antenna with a radius of ~2 mm, which is placed on a two-dimensional (2D) linear translation stage. The RF field collected by the probe antenna is then sent to an RF spectrum analyser for power measurement. The stage can scan in horizontal and vertical directions, thus measuring the 2D intensity profile of the received beam.

### The method for the measurement of the interference pattern of the generated beams with a Gaussian beam

To measure the transverse phase of an OAM beam, we use a Gaussian beam to interfere with the OAM beam, as shown in Fig. 7b. The two beams are combined using a beam splitter, which was fabricated using a printed-circuit board with a spatially varying reflective surface. It has 50% transmission efficiency at 28 GHz when the beam has a 45° incident angle^9^.

### The method for the intensity profile measurement of the generated beams with some steering angle

When the generated OAM beam is steered, we first estimate the propagation direction of the generated beam. As shown in Fig. 7c, the 2D stage is then placed 1.2 m after the circular antenna array to scan the intensity profile of the steered OAM beam. The 2D stage scans in a plane perpendicular to the estimated propagation direction of the beam to get the intensity profiles of the steered OAM beams.

### The method for the mode purity measurement

As shown in Fig. 7d, to measure the received power distribution on different modes when a specific mode is transmitted (mode purity), we first use a spiral phase plate (SPP) with the OAM order of −*ℓ* to convert the OAM +*ℓ* component of the received OAM beam into a Gaussian-like beam. A horn antenna is then followed to collect the Gaussian-like beam for power measurement. In our experiment setup, the SPP is defined by its thickness which varies azimuthally according to h(θ) = θ/2π·*ℓλ*/(*χ* − 1), acquiring a maximum thickness difference of Δh = *ℓλ*/(*χ* − 1) (θ is the azimuthal angle, *χ* is the refractive index of the plate material and λ is the wavelength of the millimeter-wave)^9^. When a specific beam is received, we sequentially apply SPPs with different orders, thereby measuring the magnitude of parasitic OAM components.

### The simulation method

We have also simulated the generation and steering of OAM beams using antenna arrays. In the simulation, we first generate the electrical field of a Gaussian beam at the distance of *z*^33^:


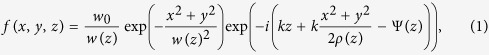


where (*x, y*) is the plane perpendicular to the transmission direction of the Gaussian beam at the distance *z*; *k* = 2; *π*/*λ* is the wave number and *λ* is the wavelength; *w*_0_ is the beam waist; 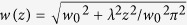; 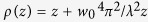; 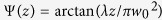. We then calculate the electrical field of the whole beam emitted from the antenna array as:


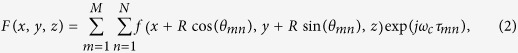


where *M* is the number of generated and steered OAM beams; *N* is the number of the antenna elements; *R* is the distance from the antenna elements to the array center; *θ*_*mn*_ = 2*π*(*n* − 1)/N is the azimuthal angle of *n*-th antenna element from a plane; and *τ*_*mn*_ is the time delay for the *n*-th branch of the *m*-th generated beam.

## Additional Information

**How to cite this article**: Xie, G. *et al*. Demonstration of Tunable Steering and Multiplexing of Two 28 GHz Data-Carrying Orbital Angular Momentum Beams Using Antenna Array. *Sci. Rep.* **6**, 37078; doi: 10.1038/srep37078 (2016).

**Publisher’s note:** Springer Nature remains neutral with regard to jurisdictional claims in published maps and institutional affiliations.

## Supplementary Material

Supplementary Information
