# Supplementary material for: Demonstration of Tunable Steering and Multiplexing of Two 28 GHz Data Carrying Orbital Angular Momentum Beams Using Antenna Array
Source: Sci Rep. 2016 Nov 11;6:37078. doi: 10.1038/srep37078 (PMC5105144; doi:10.1038/srep37078)
Supplement: Supplementary Information [file srep37078-s1.pdf]

Demonstration of Tunable Steering and Multiplexing of Two 28 GHz Data Carrying Orbital Angular Momentum Beams Using Antenna Array: Supplementary

Guodong Xie[1,*], Zhe Zhao[1], Yan Yan[1], Long Li[1], Yongxiong Ren[1], Nisar Ahmed[1], Yinwen Cao[1], Asher J. Willner[1], Changjing Bao[1], Zhe Wang[1], Cong Liu[1], Morteza Ziyadi[1], Shilpa Talwar[2], Soji Sajuyigbe[2], Solyman Ashrafi[3], Moshe Tur[4], Andreas F. Molisch[1], Alan E. Willner[1,*]

[1]Department of Electrical Engineering, U. of Southern California, Los Angeles, CA 90089, USA

[2]Intel Labs, Intel Corporation, Santa Clara, CA 95054, USA

[3]NxGen Partners, Dallas, TX 75219, USA

[4]School of Electrical Engineering, Tel Aviv University, Ramat Aviv 69978, Israel

Corresponding email: guodongx@usc.edu, willner@usc.edu

Generation efficiency and antenna number: One important factor for the generation of OAM beams using an antenna array is its generation efficiency, in terms of the ratio of the power on the most inner ring of the beam over the power of the whole beam. Figure S1a shows a few designed antenna array structures, where L is the number of concentric layers housing the antenna elements, and N_s is the number of elements, equally distributed on the circumference of each layer. By carefully designing the structure of the array, better generation efficiency could be achieved: (*i*) as the number of elements per layer increases, more power is concentrated on the inner ring, as shown in Fig. S1b1-b3; and (*ii*) increasing the number of layers could also improve the generation efficiency, as shown in Fig. S2b3-b6. When the array has four layers, each containing 32 elements, a generation efficiency of ~58.26% is observed in the simulation.

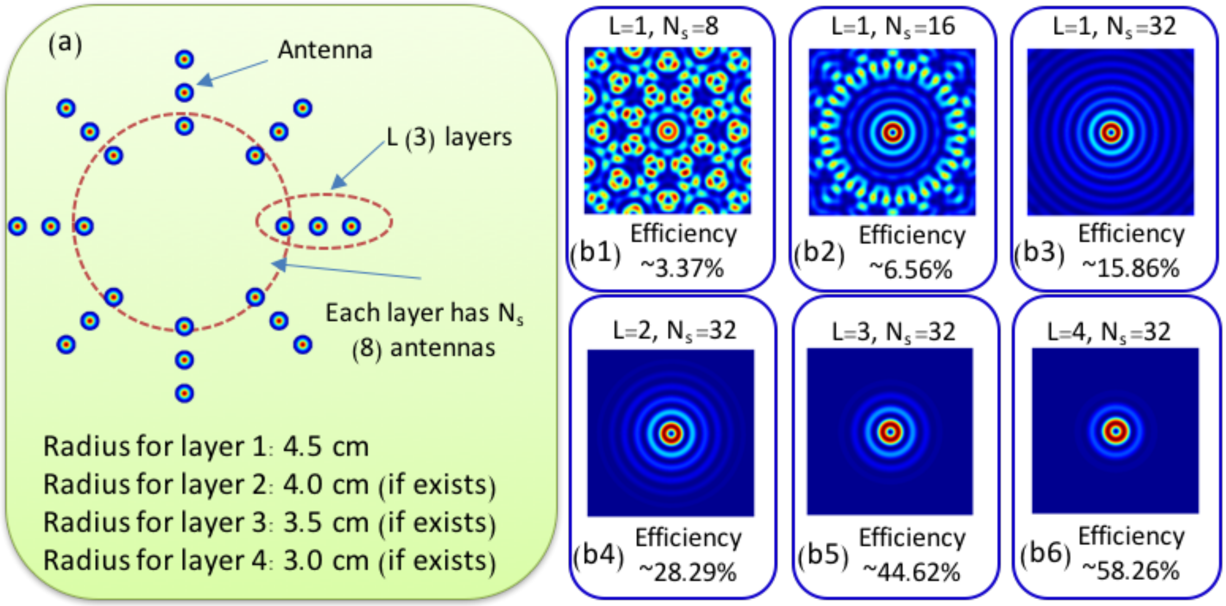

Figure S1 | Simulation Results for Different Antenna Arrangements. (a) The arrangement of the antennas; L: number of ring layers, the cases of L=1, 2, 3, 4 are investigated; N_s: number of antennas on each of the rings, the cases of N_s=8, 16, 32 are investigated. (b1-b6) The intensity profiles of the generated OAM+1 beam for different L and N_s values. Efficiency: the power on the inner-ring of the generated beam over the power of the whole beam.
